# Factors influencing early post-wildfire vegetation and implications for invasive plant management in the interior of British Columbia, Canada

**DOI:** 10.1186/s42408-026-00463-x

**Published:** 2026-03-05

**Authors:** Virginia V. Oeggerli, Tara G. Martin, Suzanne W. Simard, Jennifer Grenz

**Affiliations:** 1 Department of Forest Resources Management, University of British Columbia, 2424 Main Mall, Vancouver, BC V6T1Z4 Canada; 2 Department of Forest & Conservation Sciences, University of British Columbia, 2424 Main Mall, Vancouver, BC V6T1Z4 Canada

**Keywords:** Wildfire, Invasive plants, Native plants, Post-fire vegetation, Ecological restoration

## Abstract

**Background:**

Wildfire activity is increasingly characterized by larger fire events and a greater prevalence of high-severity burns, driven by climate change, land-use change, and prolonged fire suppression. These shifts are altering post-fire vegetation dynamics, yet uncertainty remains regarding how early post-fire plant responses vary with burn severity and pre-fire occurrence of invasive plants under contemporary fire conditions. Given limited capacity for post-fire monitoring and restoration across increasingly large fire-affected landscapes, identifying factors associated with early post-fire vegetation responses is critical for prioritizing surveillance and supporting invasive plant management through early detection and rapid response (EDRR) programs. The 46,000 ha McKay Creek Wildfire in interior British Columbia, Canada, provided an opportunity to examine how burn severity, topography, and pre-fire occurrence of invasive plants (based on mapped infestation extent) influence early post-fire vegetation composition across diverse ecosystems. We predicted that both high burn severity and pre-fire occurrence of invasive plants would be associated with increased non-native plant cover following wildfire.

**Results:**

Vegetation cover was recorded by species and grouped by native status (native or non-native), and life cycle (annual, biennial, perennial), on 80 plots stratified by burn severity and pre-fire occurrence of invasive plants. Two years post-fire, vegetation cover was dominated by bare ground across all plots, while native plant cover exceeded non-native cover under all conditions. At the broad status-group level (native vs. non-native), post-fire vegetation cover did not differ meaningfully across burn severity classes or between areas with and without documented pre-fire occurrence of invasive plants. Instead, elevation was the strongest driver of early post-fire vegetation patterns, with native cover increasing and non-native cover and bare ground decreasing at higher elevations. When vegetation was disaggregated by status and life cycle, non-native annual cover was higher in high-severity burns and at lower elevations, and non-native perennial cover increased with elevation.

**Conclusions:**

At a time when wildfires are increasing in size, frequency, and intensity, and resources for recovery are limited, this study provides region-specific insights to support prioritization of early post-fire restoration activities such as monitoring, prevention and management of invasive plants, and planting of native species.

## Resumen

**Antecedentes:**

La actividad de los fuegos de vegetación se está caracterizando por eventos de incendio cada vez más grandes y una mayor prevalencia de quemas de alta severidad, conducidas por el cambio climático, cambios en el uso de la tierra, y una prolongada era de supresión de fuegos. Estos cambios están alterando la dinámica de la vegetación en el post-fuego, y aún así, la incertidumbre permanece sobre cómo las respuestas de las plantas en el post fuego temprano varían con la severidad de la quema y con la ocurrencia de plantas invasoras en el período previo al fuego en las actuales condiciones de los incendios. Dada la limitada capacidad para monitorear en el post fuego y en la restauración a través de grandes paisajes afectados por fuegos, la identificación de factores asociados con la respuesta inicial de las plantas en el post fuego temprano es crítico para priorizar la vigilancia y el soporte del manejo de plantas invasoras a través de programas de rápida detección y respuesta rápida (EDRR). Las 46 mil ha del incendio de McKay Creek en el interior de la Columbia Británica, proveen de una oportunidad para examinar cómo la severidad de ese incendio, la topografía del lugar, y la existencia, previa al fuego, de especies invasoras (basados en mapas de extensión de la infestación), influencian la composición de la vegetación en el post fuego temprano y a través de diversos ecosistemas. Nuestra predicción fue que tanto la severidad de las quemas y la pre-ocurrencia de plantas invasoras, podría asociarse con un incremento en la cobertura de plantas invasoras (no nativas) en el post fuego temprano.

**Resultados:**

La cobertura de la vegetación fue registrada por especies y agrupadas por su estatus (Nativas y No-nativas) y ciclo de vida (anuales, bienales, y perennes), en 80 parcelas estratificadas por la severidad del fuego y la existencia previa de plantas invasoras. Dos años luego del incendio, la cobertura vegetal fue dominada por el suelo desnudo en todas las parcelas, mientras que la cobertura de plantas nativas excedió a la de no-nativas bajo todas las condiciones. A nivel de estatus amplio (entre grupos de nativas vs. no-nativas) la cobertura post fuego no difirió significativamente entre las clases de severidad o entre áreas documentadas previamente con existencias o no de especies invasoras. En cambio, la elevación del terreno fue el factor conducente más importante de los patrones de vegetación encontrados en el post fuego, con una cobertura creciente de plantas nativas y un decrecimiento tanto de plantas no nativas como de suelo desnudo a mayores elevaciones. Cuando la vegetación fue desagregada por estatus y ciclos de vida, la cobertura de anuales no-nativas fue mayor en quemas de alta severidad y a bajas elevaciones, y la cobertura de plantas perennes no-nativas se incrementó con la elevación.

**Conclusiones:**

Cuando los incendios de vegetación se incrementan en tamaño, frecuencia, e intensidad, y los recursos para la recuperación de la vegetación son limitados, este estudio provee de percepciones específicas de valor regional para priorizar actividades de restauración post fuego como el monitoreo, prevención, y manejo de plantas invasoras, y la plantación de especies nativas.

## Background

Globally, wildfire activity is changing, with increases in the frequency and severity of large wildfire events, driven by the cumulative impacts of climate change, land-use change, and prolonged fire suppression (Abatzoglou and Williams 2016; Bowman et al. 2020; Flannigan et al. 2013; Sayedi et al. 2024). As a result, vegetation dynamics are shifting across fire-prone ecosystems (Bowman et al. 2020). Although post-wildfire vegetation responses have been studied extensively, particularly in the western USA (Abella and Fornwalt 2015), evidence in the literature is more limited regarding the drivers of early post-fire vegetation responses under contemporary, high-severity wildfire conditions, especially from studies that directly link post-fire responses to documented pre-disturbance conditions (Balch et al. 2013; Flannigan et al. 2013; Parks et al. 2015; Abatzoglou and Williams 2016; Archibald et al. 2018). Existing studies suggest that vegetation responses under these conditions can be highly variable and context-dependent across ecosystems and fire conditions (Johnstone et al. 2016; Coop et al. 2020).

Wildfire can substantially alter plant communities by changing species composition, reducing vegetation cover, and disrupting regeneration processes (Fornwalt and Kaufmann 2014). High-severity fires may result in the loss of mature vegetation and soil seed banks while also modifying soil structure, moisture availability, and nutrient dynamics (Neary et al. 1999; Certini 2005; Harvey et al. 2016). Understanding early post-fire vegetation responses therefore requires consideration of species-level traits that govern persistence, reproduction, recruitment, and establishment.

Plant life-history strategies, including differences among annual, biennial, and perennial species, play a key role in shaping how species respond to disturbance and recovery over time (Dixon et al. 2024, Ricci et al. 2024; Shryock et al. 2014). Trait-based syntheses show that disturbance responses are broadly predictable based on plant life-history strategies, with short-lived, seed-bank–dependent species consistently favored over long-lived perennials that rely on belowground persistence (Dixon et al. 2024). Annual and biennial species are often favored immediately after fire due to short life cycles, rapid growth, and high production of small, readily dispersed seeds, and may include a high proportion of invasive plants when propagules are available from surrounding landscapes (Ricci et al. 2024). Studies of post-fire understory succession show that short-lived forbs increase in richness and cover following moderate to high-severity fire, whereas long-lived perennial species recover more gradually and contribute to longer-term community structure and stability (Fornwalt and Kaufmann 2014). Longer-term resilience in post-fire plant communities is therefore mediated by perennial species whose persistence and recovery shape community stability over time (Shryock et al. 2014; Day et al. 2017).

Early post-fire patterns can also influence longer-term community development by shaping initial dominance, propagule availability, and fuel conditions, which may contribute to persistent compositional change or homogenization documented in later post-fire intervals (Weeks et al. 2023; Abella and Fornwalt 2015). Accordingly, identifying drivers of early post-fire responses can help target monitoring and intervention during a period when management actions may have a disproportionate influence on subsequent ecosystem development.

Many of the species-level traits associated with early post-fire establishment—including dispersal attributes that facilitate swift colonization of disturbed areas—also underlie the success of invasive plants. Invasive plants, generally defined as a non-native species to a particular ecosystem that is causing some degree of harm to humans and/or the environment (Richardson and Pysek 2004, NISC 2005), represent an additional, globally relevant factor influencing post-wildfire vegetation composition as their competitive characteristics, combined with shifting fire regimes, may create opportunities for establishment and dominance in habitats where they were previously limited (Zouhar et al. 2008; Alba et al. 2015). Invasive plants often exhibit traits such as high reproductive output, effective dispersal, broad environmental tolerances, and phenotypic plasticity that can allow them to respond more readily to climatic changes and disturbance than many native species (Dukes et al. 2009; Birthisel et al. 2021; Shephard et al. 2022; Jones and Grenz 2023). These traits can confer competitive advantages in fire-disturbed environments (Richardson and Pyšek 2004; Dukes et al. 2009; Alba et al. 2015). In addition to ecological mechanisms, wildfire suppression and response activities can facilitate human-mediated dispersal of invasive plants through fire-line and fire-guard construction that increases exposed mineral soil, and the movement of potentially contaminated equipment, vehicles, and materials during fire control operations and post-fire rehabilitation, further increasing invasion risk following wildfire (Keeley 2006; Merriam et al. 2006).

When invasive plants establish or expand following wildfire, they may alter fuel structure, continuity, and flammability, increasing the likelihood of subsequent fires and creating positive fire–invasion feedback loops (D’Antonio and Vitousek 1992; Brooks et al. 2004; Balch et al. 2013). Such feedbacks have been documented across diverse ecosystems globally, where invasive grasses and forbs have altered fire regime characteristics by increasing fire frequency or intensity, often at the expense of fire-resilient native species adapted to different fire patterns (Chambers et al. 2014; Grenz and Clements 2023).

Despite increasing recognition of the factors shaping post-wildfire vegetation responses, empirical studies in British Columbia’s southern interior remain limited (Dickson-Hoyle et al. 2024). This region includes dry forests, grasslands, and complex topography, where fire regimes have shifted markedly from historical patterns shaped by Indigenous fire stewardship over millennia (Heyerdahl et al. 2012; Lewis et al. 2018). Colonization-era fire exclusion, industrial logging, grazing, and climate warming have collectively altered fuel structures and vegetation composition, increasing the likelihood of large, high-severity wildfires (Hagmann et al. 2013, 2021; Harvey et al. 2017). These trends mirror a broader global acceleration of fire regime change over the past two centuries (Sayedi et al. 2024). In British Columbia, the 2023 fire season alone burned 2.84 million ha, surpassing previous records and underscoring the growing prevalence of large, high-severity wildfire events (Canadian Interagency Forest Fire Centre (CIFFC) 2024, Government of British Columbia 2024).

Post-fire vegetation dynamics in British Columbia’s interior are further complicated by the widespread occurrence of invasive non-native plant species. Species such as *Centaurea stoebe* (spotted knapweed) and *Bromus tectorum* (cheatgrass) are well established across many dry forest and grassland systems, particularly along anthropogenically disturbed corridors (e.g., powerlines) and grazed rangelands (Leung 2002, Invasive Species Council of BC 2024a, 2024b).

Although fire-invasion dynamics have been documented globally, uncertainty remains regarding the relative importance of different drivers influencing post-fire vegetation responses across ecosystems and fire contexts (Alba et al. 2015; Johnstone et al. 2016). In particular, the roles of burn severity and documented pre-fire occurrence of invasive plants warrant closer examination, especially in landscapes increasingly characterized by high-severity wildfire. Previous studies indicate that non-native plants often respond differently to wildfire than native species, emphasizing the need to evaluate factors that may account for these divergent responses (Alba et al. 2015). In British Columbia’s interior, where high-severity burns now comprise a greater proportion of burned area than under historical fire regimes (Heyerdahl et al. 2012; Collins et al. 2025), understanding these drivers is critical for anticipating vegetation change and informing restoration planning.

We focused on the McKay Creek Wildfire, a 46,000 ha fire that burned near Lillooet, British Columbia, igniting in June 2021 during the historic heat dome. This fire provided an uncommon opportunity to examine early post-fire vegetation responses because prior to the fire, the study area had been systematically surveyed for the occurrence and extent of priority invasive plants as part of coordinated monitoring and control efforts led by the regional invasive species committee in collaboration with the BC Provincial Invasive Plant Program and local Indigenous communities. The availability of these pre-fire baseline data created an opportunity to evaluate how burn severity and pre-existing occurrence of invasive plants may influence vegetation composition following wildfire. The study was motivated by the need to support early detection and rapid response (EDRR) by identifying areas at higher risk of early post-fire invasion and informing timely, targeted restoration and invasive plant interventions (Inter-Ministry Invasive Species Working Group [IMISWG] 2014).

In this study, we assessed vegetation composition 2 years after the McKay Creek Wildfire across a diverse landscape spanning four Biogeoclimatic Ecosystem Classification (BEC) zones (British Columbia Ministry of Forests n.d., MacKenzie and Meidinger 2018), diverse topographical features (aspect, slope, elevation), and three burn severity classes (low, medium, and high). While multiple environmental variables were considered, our analysis prioritizes burn severity and pre-fire occurrence of invasive plants as potential key factors influencing post-fire vegetation responses.

Vegetation responses were evaluated using standard plant classification frameworks commonly employed in post-fire ecology to facilitate comparison with the broader fire ecology literature. Although related analyses from the same landscape context showed that culturally informed plant groupings can yield different interpretive insights (Oeggerli and Grenz 2025), the present study adopts conventional classifications to align with established fire ecology literature and support cross-study synthesis, while recognizing that standard categories do not explicitly account for Indigenous knowledge and value systems (Armstrong et al. 2025).

Given the increasing scale of wildfire disturbance across British Columbia’s interior, our overarching research question was which factors most strongly influence early post-fire native and non-native plant responses in ways that can inform invasive plant monitoring, prevention, and control, as well as post-fire restoration planning. Specifically, we addressed two research questions: (1) how is burn severity associated with early post-wildfire vegetation cover, and (2) is pre-fire occurrence of invasive plants associated with early post-wildfire vegetation cover? We predicted that both pre-fire occurrence of non-native invasive plants and high burn severity would be associated with increased non-native plant cover in this early post-fire landscape. Rather than testing long-term successional trajectories, this study evaluates early post-fire vegetation patterns to examine how disturbance severity and pre-existing invasion influence short-term responses. Overall, our objective was to identify factors associated with early post-fire vegetation responses to support data-driven post-wildfire restoration and invasive plant management, including prevention and control of invasive plants, and revegetation of native species.

## Methods

### Study area

The McKay Creek Wildfire ignited on June 29, 2021, in British Columbia’s southern interior, and was determined to be human caused. The fire started during a period of extreme heat and elevated fire weather conditions associated with the 2021 Pacific Northwest heat dome (Still et al. 2023). Rapid fire growth during the initial days of the event, with periods of increased fire activity in August, and continued burning into September, prompted sustained suppression efforts by the British Columbia Wildfire Service over multiple weeks. Fire behavior varied across complex terrain, resulting in extensive contiguous areas of high-severity burn interspersed with patches of low- and medium-severity burn. The fire occurred within the traditional territory of the St’át’imc Nation, specifically the traditional territories of six Northern St’át’imc communities, Sekw’el’was, T’ít’q’et, Tsal’alh, Ts’kw’aylaxw, Xaxli’p, and Xwísten.

**Fig. 1:**
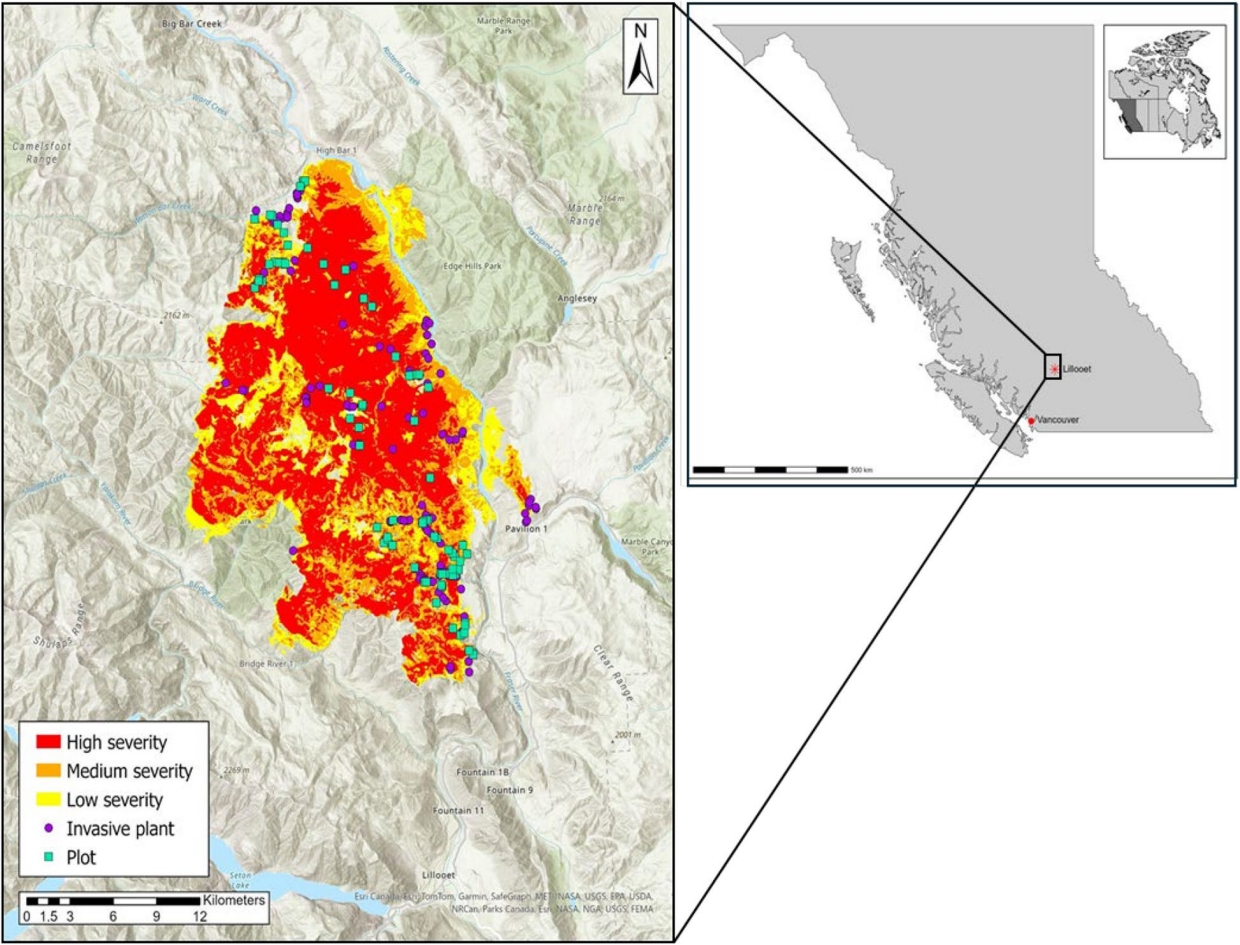
Map of the McKay Creek Wildfire in British Columbia’s southern interior, Canada, showing the fire perimeter, burn severity classes, vegetation sampling plot locations, and locations of previous invasive plant surveys. Burn severity was classified using satellite-derived differenced Normalized Burn Ratio (dNBR) and categorized into severity classes following the Burned Area Reflectance Classification (BARC) system. Plot locations represent discrete sampling sites distributed across the wildfire footprint

The study area spanned the McKay Creek Wildfire footprint (Fig. 1), which includes major drainages of the Fraser River, Bridge River, and Seton Lake. This region is characterized by a warm, dry climate, average daytime high and low seasonal temperatures of 25.9 °C and 12.6 °C in the summer, and 4 °C and −3.6 °C in winter, approximately 31 days per year of temperatures exceeding 30 °C, and annual precipitation of approximately 349 mm (Environment and Climate Change Canada 2024). It has steep, rugged terrain with narrow valleys and high ridges. Elevations within the McKay Creek wildfire ranged from approximately 400 to 1800 m, and this pronounced elevation gradient contributes to the region’s highly variable microclimates and diverse ecosystems. The fire spanned four Biogeoclimatic Ecosystem Classification (BEC) zones: bunchgrass (dry, low-elevation grasslands occurring on benches and slopes, and dominated by perennial grasses, forbs, and shrub cover), ponderosa pine (warm, dry low-elevation forests characterized by open canopies and grass-dominated understories), interior Douglas-fir (mesic mid-elevation forests with closed to semi-open canopies and well-developed shrub and grass layers), and montane spruce (cool, high-elevation forests with relatively closed canopies and sparse to moderately developed understories dominated by low-growing forbs and dwarf shrubs), ranging from low-elevation grasslands to high-elevation subalpine forests (Meidinger and Pojar 1991). The study area includes areas with a history of anthropogenic disturbance, most notably forestry and cattle grazing, that were present on the landscape prior to the 2021 wildfire. These disturbances are evident in features such as cut blocks, access roads, and grazed grassland and forest understories within the fire perimeter and provide context for the occurrence of invasive plants documented in the study area.

### Field sampling design

Within the McKay Creek Wildfire area, 80 9-m^2^ (3 m × 3 m) vegetation monitoring plots were established using a combination of preferential and stratified random sampling methods (Michalcová et al. 2011). Preferential plot placement was informed through consultation with the T’ít’q’et, Ts’kw’aylaxw, and Xwísten communities to ensure sampling included areas of local concern, while still meeting the stratification criteria. Plots were stratified based on two key ecological factors: burn severity (low, medium, high) and prior invasive plants occurrence (yes/no). Burn severity classes were derived from satellite-based burn severity mapping produced by the British Columbia Ministry of Forests, Forest Analysis and Inventory Branch, using differenced Normalized Burn Ratio (dNBR) calculated from pre- and post-fire Landsat or Sentinel-2 imagery and classified following the Burned Area Reflectance Classification (BARC) system (British Columbia Ministry of Forests 2021). Prior occurrence of invasive plants was determined using records from British Columbia’s Invasive Alien Plant Program and provided for the study area by the Lillooet Regional Invasive Species Society (LRISS). Although invasive plant surveys in the region extend further back in time, only records collected within the 20 years preceding the wildfire were used. Only invasive plants identified as priority plants by the Province of British Columbia were included in this study (British Columbia Ministry of Forests 2025). Survey records include georeferenced point observations and mapped infestation polygons, with associated information on infestation area, while density and spatial distribution are recorded using predefined categorical classes following provincial standards (British Columbia Ministry of Forests and Range 2010). For stratification purposes, records representing infestations of ≥3 m^2^ were retained and simplified to a binary indicator of occurrence of invasive plants (presence or absence).

Using the three burn severity classes (low, medium, high) and presence or absence of invasive plant, the plots were stratified into six unique treatments. We established 13 3 m × 3 m plots in each strata, except for the “low yes” treatment, which included 15 plots.

Plot locations for each treatment combination were generated using the Create Random Points tool in ArcGIS Pro. Random points were generated within polygons corresponding to each of the six stratification categories defined by burn severity and prior occurrence of invasive plants; these polygons were predefined to incorporate all logistical, safety, and exclusion criteria. These predefined criteria were incorporated due to the large size (46,000 ha) of the McKay Creek Wildfire and the extreme nature (steep, loose slopes and cliffs) of its terrain. All plots were located within 500 m of an access point by road to ensure researchers could reach the plots for monitoring within a reasonable timeframe. Post-wildfire salvage areas and roads were given a 30-m buffer and riparian areas were given a 50-m buffer from vegetation monitoring plots to limit external influences on the plant community composition. To stratify areas by prior occurrence of invasive plants, a 100-m buffer was applied between areas with prior occurrence and areas without prior occurrence to ensure clear separation.

### Data collection

**Fig. 2:**
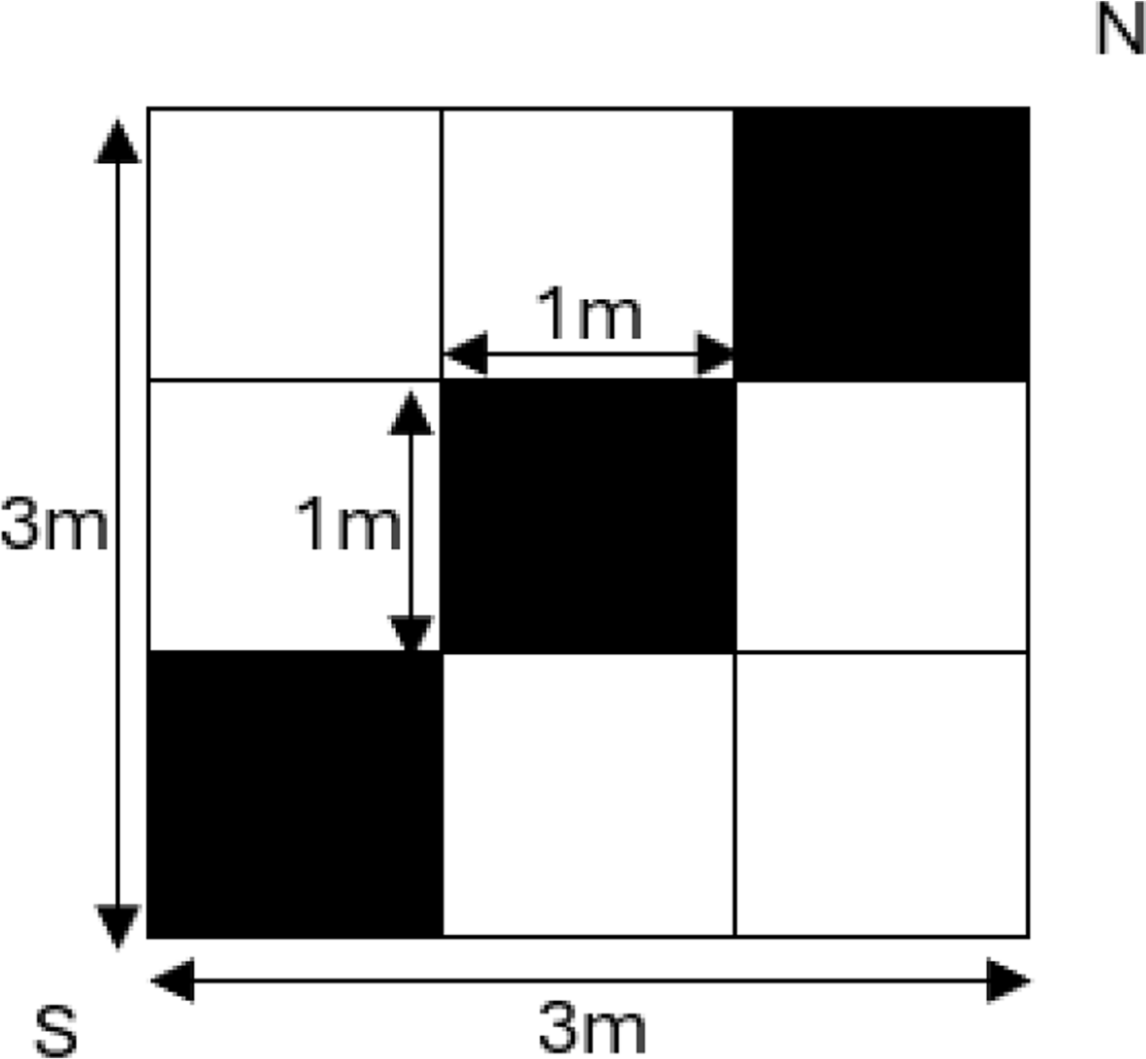
Plot sampling design. Vegetation and bare ground cover were sampled in the black squares

Within each plot, three 1-m^2^ quadrats were monitored along the north to south diagonal (Fig. 2). In each quadrat, each species was identified and percent foliar and bare ground cover recorded. Species were identified to the lowest possible taxonomic level in the field. For taxa that are difficult to reliably distinguish without reproductive structures or require specialist confirmation, specifically *Epilobium* (willowherbs), *Hieracium* (yellow hawkweeds), and *Carex* (sedges), identifications were recorded at the genus level to ensure consistency and avoid misclassification. Data collection occurred between June 15 and July 15, 2023, near or at the time of peak vegetative biomass (Applestein et al. 2018), 2 years post-fire. Plots at lower elevations with south-facing slopes were sampled first, working towards north-facing, high-elevation plots at the end of the monitoring period to ensure sites were surveyed during a period of optimal plant condition above ground.

### Percent cover plant groupings

Overall percent cover of each species in each plot was determined by averaging the three quadrats. Plant species were grouped into native (N) or non-native (NN) categories using Electronic atlas of the Flora of British Columbia (E-Flora BC) (Klinkenberg, n.d.). Native and non-native groupings were further categorized by life cycle: annual (A), biennial (B), and perennial (P) using E-Flora BC, Integrated Taxonomic Information System (ITIS), and Fire Effects Information System (FEIS) (Integrated Taxonomic Information System [ITIS] n.d., Klinkenberg n.d., U.S. Department of Agriculture, Forest Service n.d.). The individual plant species that fell within these categories were summed to provide the total percent cover for each combination of status and life cycle within a plot.

Non-native plants include all species not indigenous to the study region, regardless of their ecological impact. Invasive plants refer to a subset of non-native species designated as priority species under provincial invasive plant programs due to documented or potential ecological harm. Priority invasive plants were relatively rare and occurred at low abundance within plots during the early post-fire period; therefore, post-fire vegetation responses were analyzed using broader non-native plant groupings. Prior occurrence of invasive plants was used as a spatial risk indicator based on mapped infestations rather than as a post-fire response category.

### Statistical analysis

#### Vegetation trajectory models

We evaluated a constrained set of candidate generalized linear mixed models (GLMMs) using the glmmTMB package (Brooks 2017, Magnusson 2017) specifying a beta distribution with a logit link to assess the effects of burn severity, prior occurrence of invasive plants, and topographic variables on post-wildfire vegetation cover. Burn severity was included in all candidate models as a core predictor, while prior occurrence of invasive plants and topographic variables (elevation, slope, and aspect) were evaluated in alternative combinations to assess their associations with vegetation cover. Elevation and slope spanned broad, overlapping ranges across all burn severity and prior invasive occurrence treatments. Aspect was treated as a categorical variable using predefined cardinal and intercardinal directions (N, NE, E, SE, S, SW, W, NW).

Model comparison using Akaike’s Information Criterion (AIC) was used to assess relative support among candidate model structures, with models within ΔAIC ≤ 2 considered similarly supported. AIC rankings were used to compare alternative combinations of predictors; however, final model selection emphasized alignment with the research question by evaluating the effects of burn severity and prior occurrence of invasive plants, rather than selecting models based solely on small differences in AIC. The direction of effects for these core predictors was consistent across candidate models.

Model assumptions and fit were evaluated using simulation-based residual diagnostics in DHARMa (Hartig 2022), including residual plots (simulateResiduals), and formal tests of dispersion (testDispersion), zero-inflation (testZeroInflation), residual uniformity (testUniformity), and outliers (testOutliers). All selected models showed no evidence of non-uniform residuals, overdispersion, or influential outliers.

All analyses were conducted in RStudio (version 4.4.0). Model formulas for the selected models are provided in Table 1.

**Table 1:** Model formulas selected by plant grouping

Plant grouping	Formula
Native plant cover	Native cover (N) ~ burn severity + prior invasive occurrence + slope + elevation + (1|RE1)
Non-native plant cover	Non-native cover (NN) ~ burn severity + prior invasive occurrence + elevation + (1|RE1)
Bare ground	Bare ground ~ burn severity + prior invasive occurrence + elevation + (1|RE1)
Non-native annual cover	Non-native annual cover (NNA) ~ burn severity + prior invasive occurrence + slope + elevation + (1|RE1)
Non-native perennial cover	Non-native perennial cover (NNP) ~ burn severity + slope + elevation + aspect + (1|RE1)
Native perennial cover	Native perennial cover (NP) ~ burn severity + prior invasive occurrence + elevation + (1|RE1)

## Results

Contrary to our prediction that both pre-fire occurrence of non-native invasive plants and high burn severity would be associated with increased non-native plant cover on this early post-fire landscape, we found that pre-fire occurrence of invasive plants and high burn severity were not associated with increased non-native plant cover in this context. Instead, our results suggest that topographical factors have the most significant effects on post-wildfire vegetation responses 2 years after fire.

### Percent cover

#### Percent cover of native plant species

**Fig. 3:**
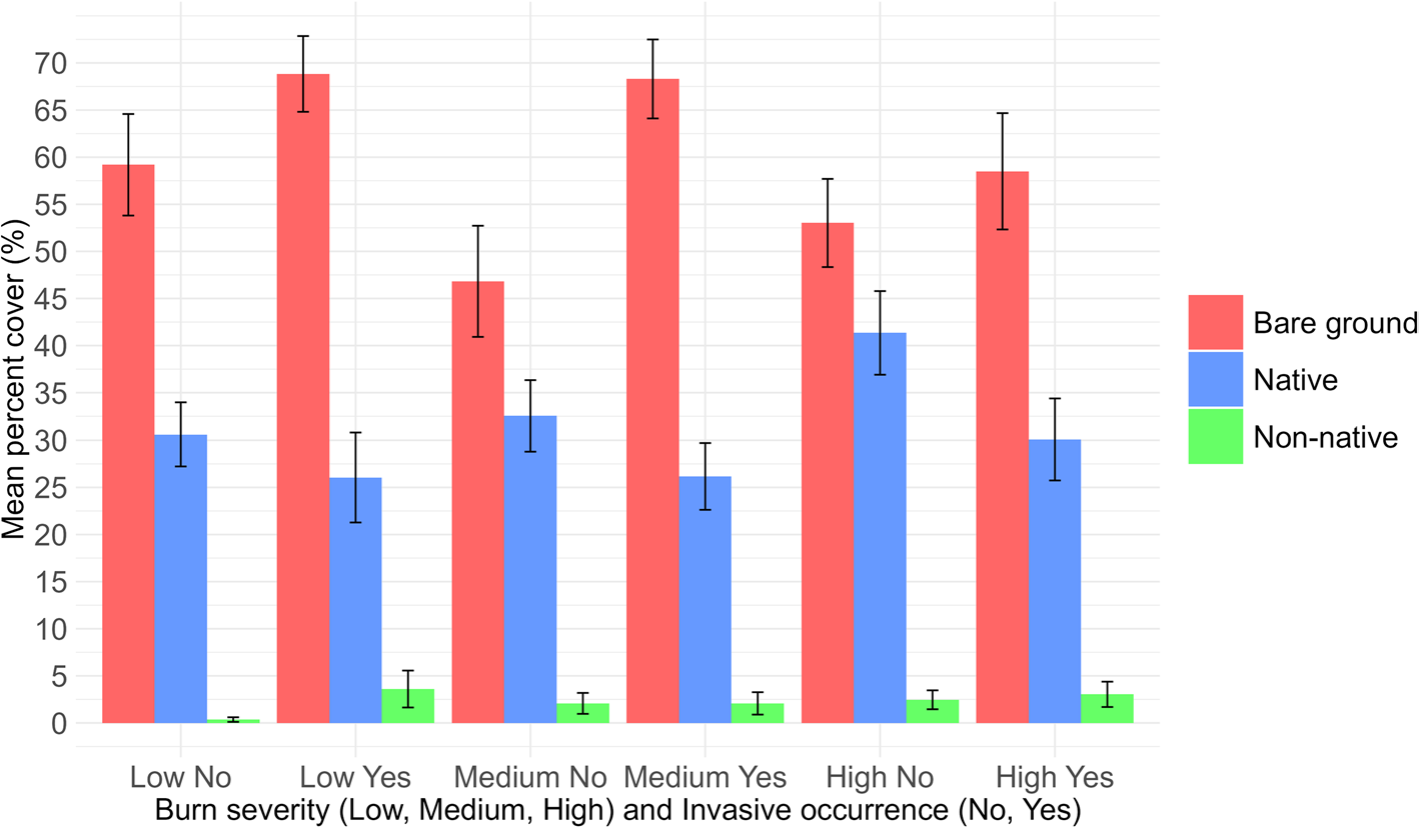
Mean cover of native plants, non-native plants, and bare ground across burn severity classes and prior invasive plants' occurrence. Bars represent mean percent cover ± standard error

Percent cover of native plant species varied with both burn severity and prior occurrence of invasive plants (Fig. 3). In the plots without prior occurrence of invasive plants prior to wildfire, the highest percent cover of native plants occurred within high burn severity plots (mean 32.56%, SE = 3.77), followed by low burn severity plots (mean 30.60%, SE = 3.38) and medium burn severity plots (mean 26.05%, SE = 4.78). In the plots with prior occurrence of invasive plants, the highest percent cover of native plants occurred within medium burn severity plots (mean 41.35%, SE = 4.44), followed by low burn severity plots (mean 26.17%, SE = 3.54) and high burn severity plots (mean 30.06%, SE = 4.32).

#### Percent cover of non-native species

The percent cover of non-native plant species was consistently low across all burn severities, regardless of prior invasive plants occurrence (Fig. 3). In the plots without prior occurrence of invasive plants prior to wildfire, the highest percent cover of non-native plant species occurred within medium burn severity plots (mean 3.60%, SE = 1.96), while low burn severity plots had the lowest percent cover (mean 0.36%, SE = 0.25). In the plots with prior occurrence of invasive plants prior to wildfire, the highest percent cover of non-native plant species also occurred within medium burn severity plots (mean 2.46%, SE = 1.00), with similar levels of percent cover in low burn severity plots (mean 2.08%, SE = 1.19) and high burn severity plots (mean 3.04%, SE = 1.34).

#### Percent cover of bare ground

The percent cover of bare ground represented almost or more than half of the percent cover across all strata (Fig. 3). In the plots without prior occurrence of invasive plants prior to wildfire, the highest percent cover of bare ground occurred within medium-severity burn plots (mean 68.83%, SE = 4.02), followed by low-severity burn plots (mean 59.19%, SE = 5.39) and high-severity burn plots (mean = 46.82%, SE = 5.90). In plots with prior occurrence of invasive plants, percent cover of bare ground was highest within low burn severity plots (mean 68.30%, SE = 4.19), followed by high-severity burn plots (mean 58.50%, SE = 6.17) and medium-severity burn plots (mean 53.01%, SE = 4.68).

### Vegetation cover models

**Fig. 4:**
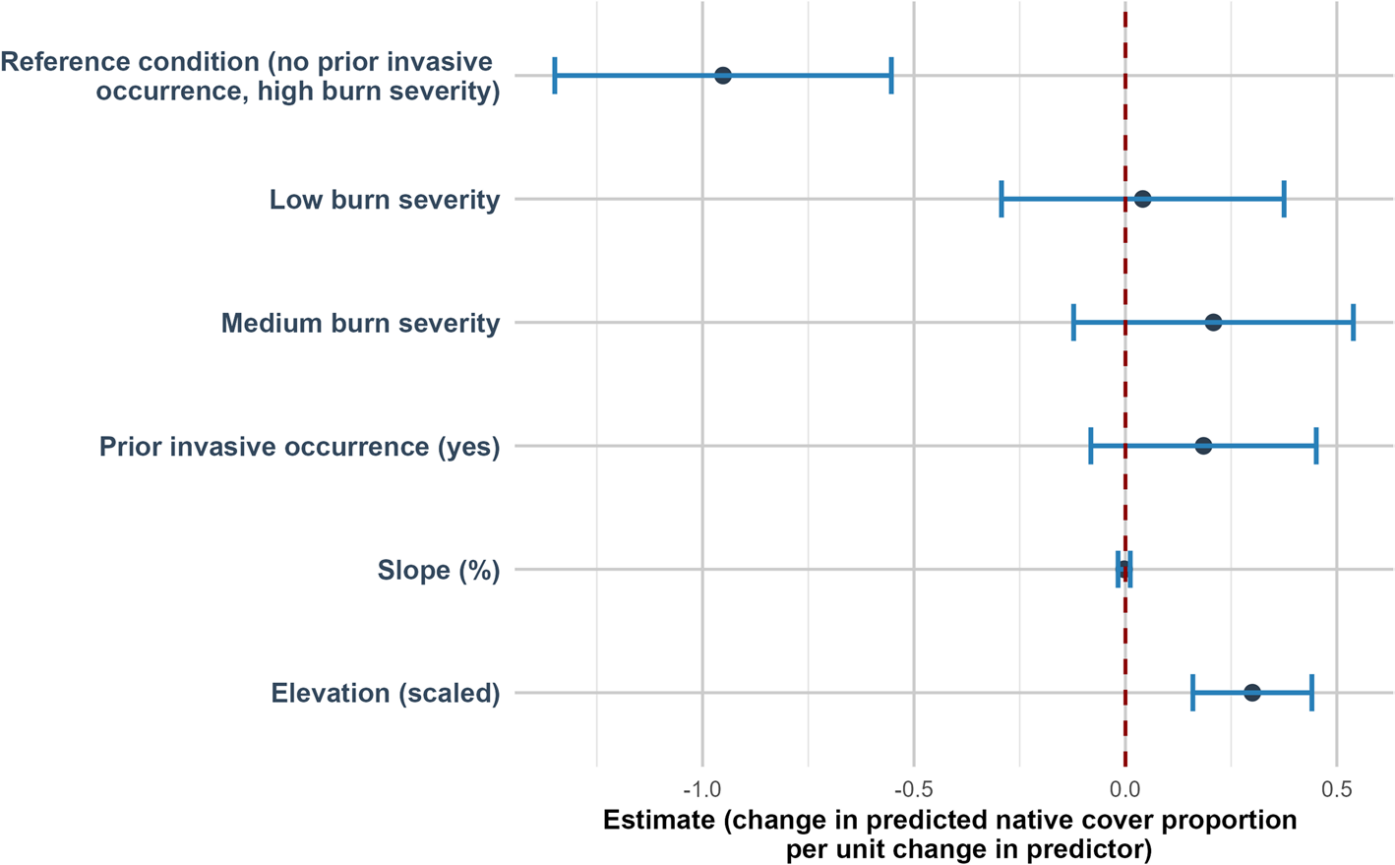
Direction and uncertainty of predictor effects on native plant cover based on generalized linear mixed models. The reference condition represents predicted native plant cover under high burn with no prior invasive occurrence, at mean-centered values of continuous predictors, and is shown for context only. Points indicate estimated model coefficients and horizontal bars represent 95% confidence intervals. Confidence intervals that do not overlap the vertical dashed zero line indicate statistically significant support for an effect, whereas intervals overlapping zero indicate weaker support

**Fig. 5:**
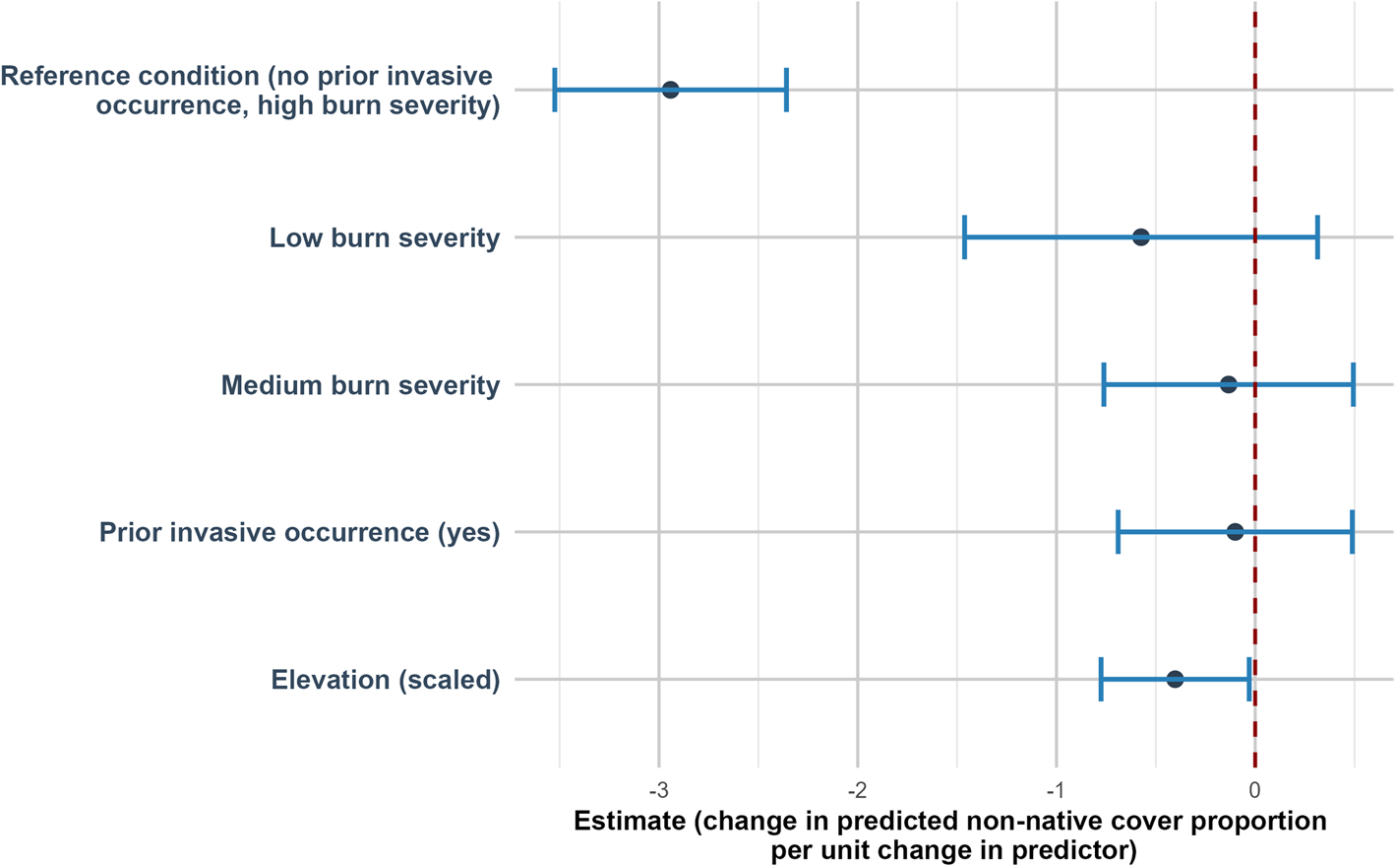
Direction and uncertainty of predictor effects on non-native plant cover based on generalized linear mixed models. The reference condition represents predicted non-native plant cover under high burn severity with no prior invasive occurrence, at mean-centered values of continuous predictors, and is shown for context only. Points indicate estimated model coefficients and horizontal bars represent 95% confidence intervals. Confidence intervals that do not overlap the vertical dashed zero line indicate statistically significant support for an effect, whereas intervals overlapping zero indicate weaker support

**Fig. 6:**
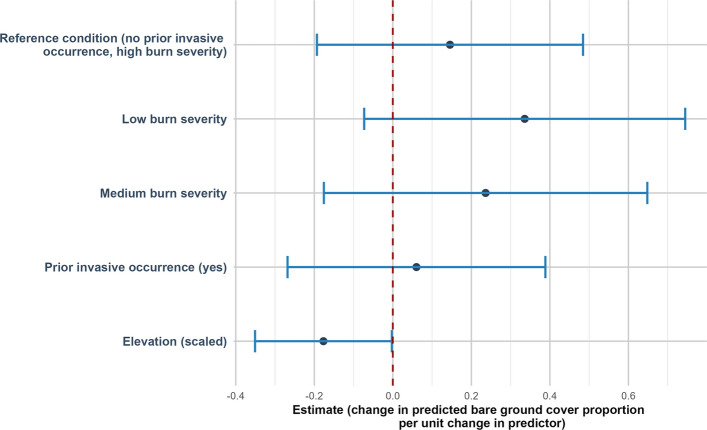
Direction and uncertainty of predictor effects on bare ground cover based on generalized linear mixed models. The reference condition represents predicted bare ground cover under high burn severity with no prior invasive occurrence, at mean-centered values of continuous predictors, and is shown for context only. Points indicate estimated model coefficients and horizontal bars represent 95% confidence intervals. Confidence intervals that do not overlap the vertical dashed zero line indicate statistically significant support for an effect, whereas intervals overlapping zero indicate weaker support

Native plant cover was positively related to elevation (estimate of 0.30 (SE = 0.07, *p* < 0.001)) (Fig. 4). Non-native plant cover was negatively related to elevation (estimate of −0.40 (SE = 0.19, *p* = 0.034)) (Fig. 5). Bare ground was negatively related to elevation (estimate of −0.18 (SE = 0.09, *p* = 0.047)) (Fig. 6).

### Vegetation cover of non-native and native plants by life cycle

Examining the mean percent cover of non-native and native plant species by plant life cycle under each treatment (no prior occurrence of invasive plants and burn severity) illustrated variation between treatments and functional plant groupings. Under no prior occurrence, non-native biennial and perennial plants showed similar cover at high burn severity, annual and biennial similar cover at medium burn severity with little perennial representation, and only biennial non-natives in the cover at low burn severity. Under no prior occurrence, perennial native plant cover was highest at high burn severity and was similar at medium and low burn severities. There were no annual or biennial native plants present at plots with no prior occurrence of invasive plants.

**Fig. 7:**
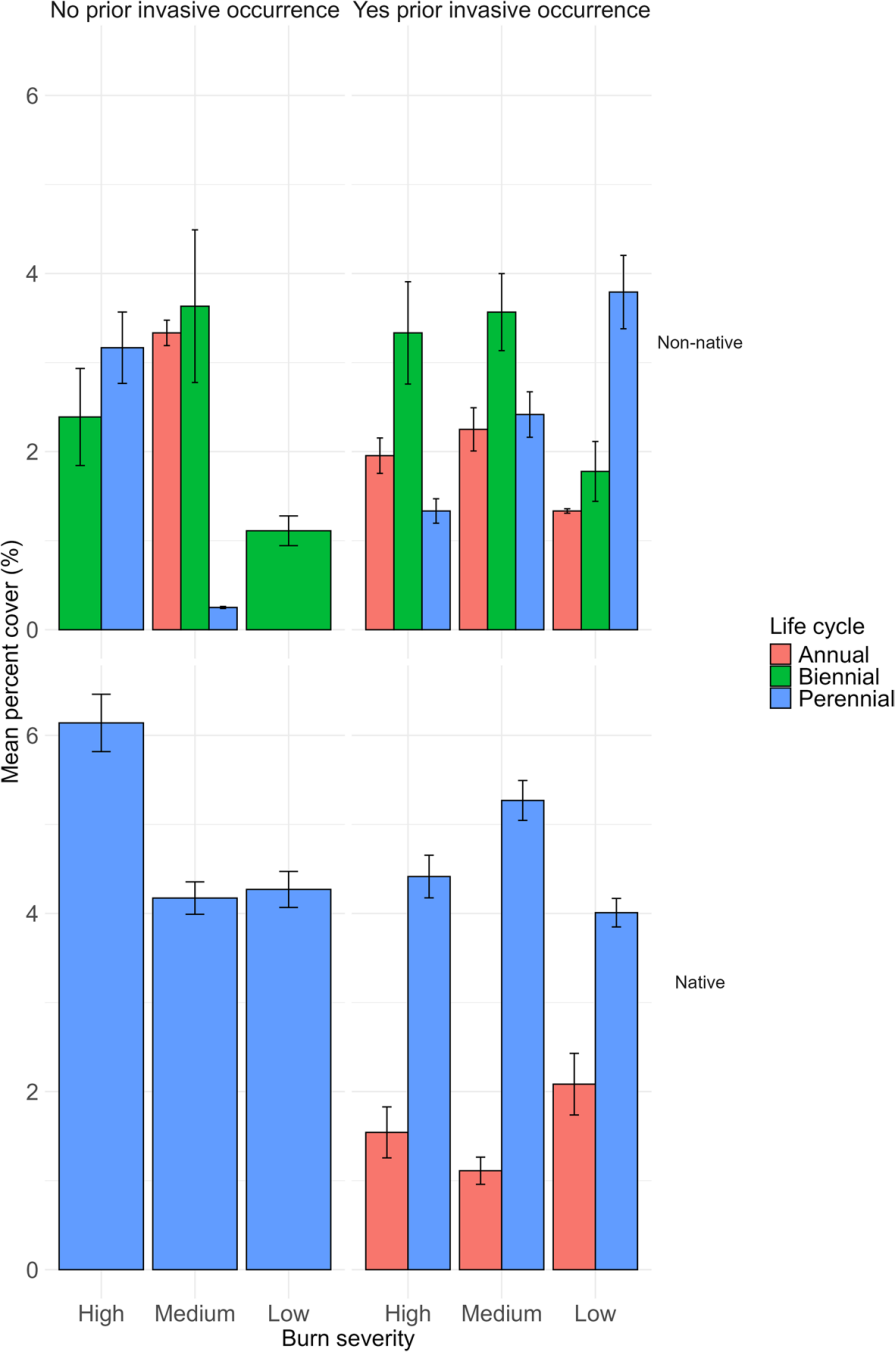
Mean cover of native and non-native plants aggregated by life cycles (annual, biennial, perennial) across burn severity classes (high, medium, low) and prior invasive plants occurrence (no, yes). Bars represent mean percent cover ± standard error

Under prior occurrence of invasive plants, non-native biennial plants had the highest mean percent cover at high and medium burn severities, with annuals and perennials having similar cover. At low burn severity, non-native perennial plants had the highest observed mean percent cover, with annuals and biennials having similar, low mean percent cover. For native species, there were no biennial species for any burn severities; perennial native plants accounted for the majority of mean percent cover at all three burn severities (Fig. 7).

### Percent cover of non-native annual plants

**Fig. 8:**
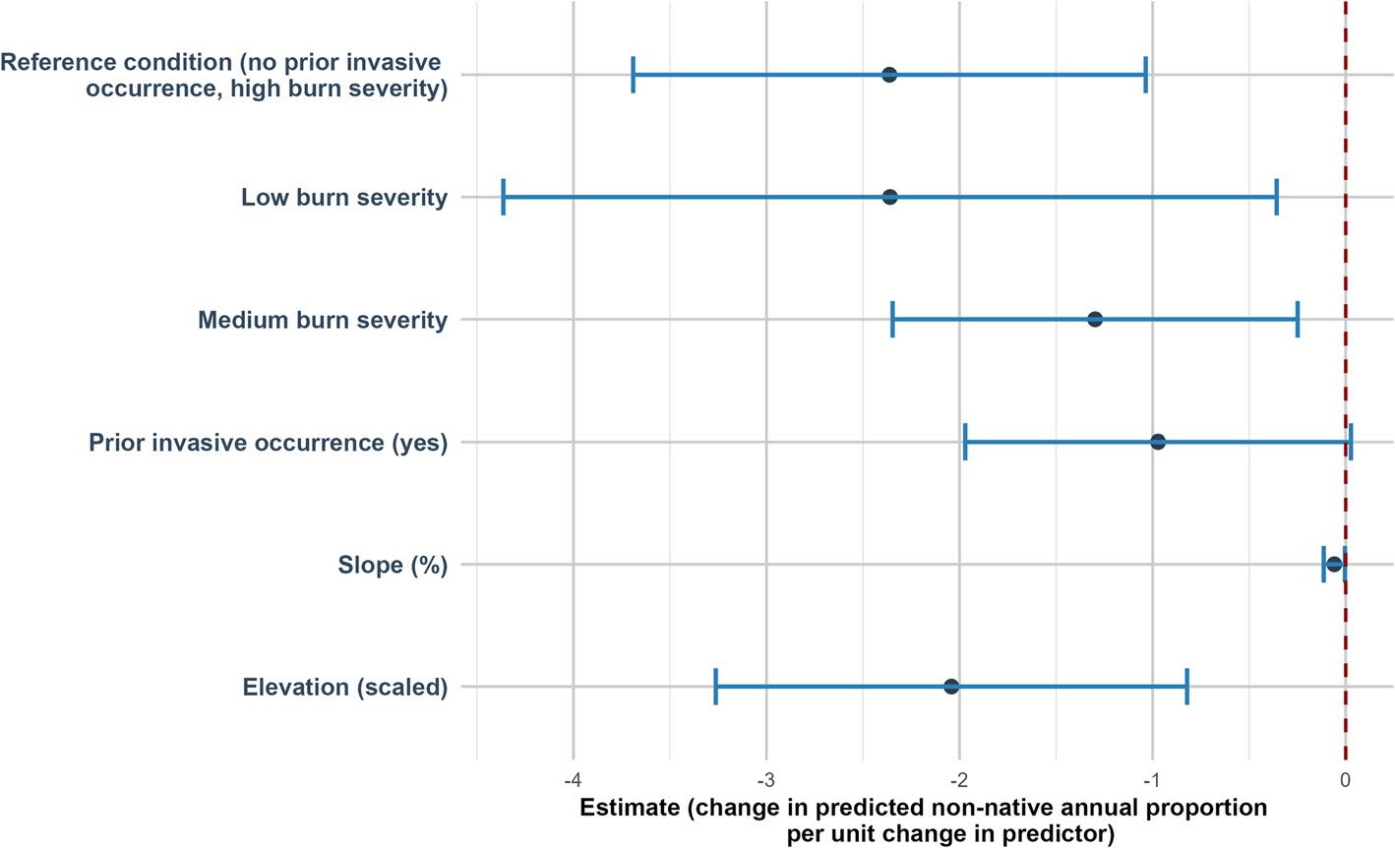
Direction and uncertainty of predictor effects on non-native annual cover based on generalized linear mixed models. The reference condition represents predicted non-native annual cover under high burn with no prior invasive occurrence, at mean-centered values of continuous predictors, and is shown for context only. Points indicate estimated model coefficients and horizontal bars represent 95% confidence intervals. Confidence intervals that do not overlap the vertical dashed zero line indicate statistically significant support for an effect, whereas intervals overlapping zero indicate weaker support

Compared to high burn severity, non-native annual (NNA) cover was significantly lower in low burn severity (estimate of −2.36 (SE = 1.02, *p* = 0.021)) and in medium burn severity (estimate of −1.30 (SE = 0.54, *p* = 0.015)). NNA cover was negatively related to slope (estimate of −0.059 (SE = 0.03, *p* = 0.035)). NNA cover was negatively related to elevation (estimate = −2.04 (SE = 0.62, *p* = 0.001)) (Fig. 8).

### Percent cover of non-native perennial plants and native perennial plants

**Fig. 9:**
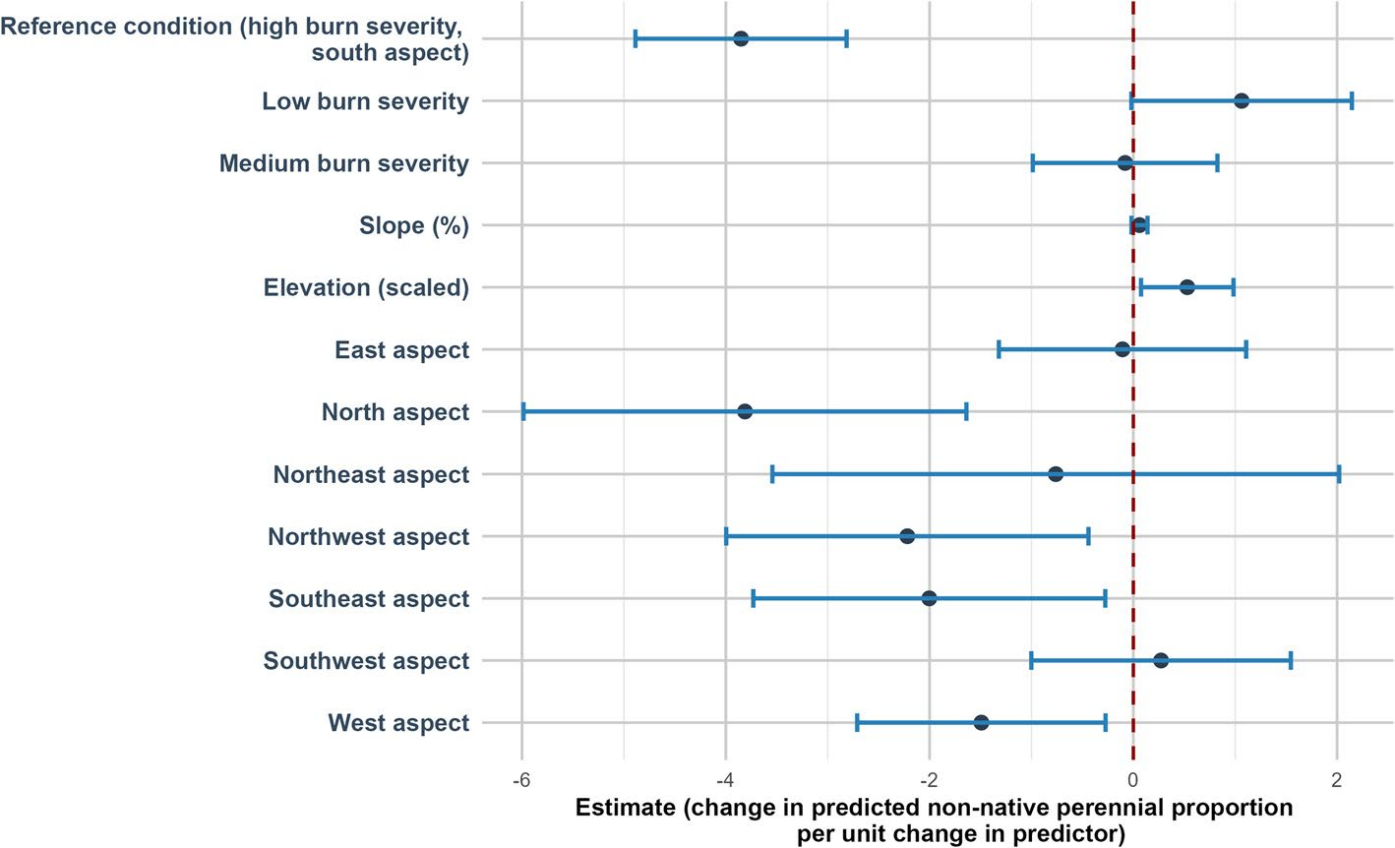
Direction and uncertainty of predictor effects on non-native perennial cover based on generalized linear mixed models. The reference condition represents predicted non-native perennial cover under high burn with no prior invasive occurrence, at mean-centered values of continuous predictors, and is shown for context only. Points indicate estimated model coefficients and horizontal bars represent 95% confidence intervals. Confidence intervals that do not overlap the vertical dashed zero line indicate statistically significant support for an effect, whereas intervals overlapping zero indicate weaker support

Non-native perennial (NNP) was positively related to elevation (estimate of 0.53 (SE = 0.23, *p* = 0.02)). Compared to south-facing aspects, NNP cover was significantly lower on north-facing (estimate of −3.81 (SE = 1.11, *p* < 0.001)), northwest-facing (estimate of −2.22 (SE = 0.91, *p* = 0.14)), southeast-facing (estimate of −2.00 (SE = 0.88, *p* = 0.023)), and west-facing aspects (estimate of −1.49 (SE = 0.62, *p* = 0.016)) (Fig. 9).

**Fig. 10:**
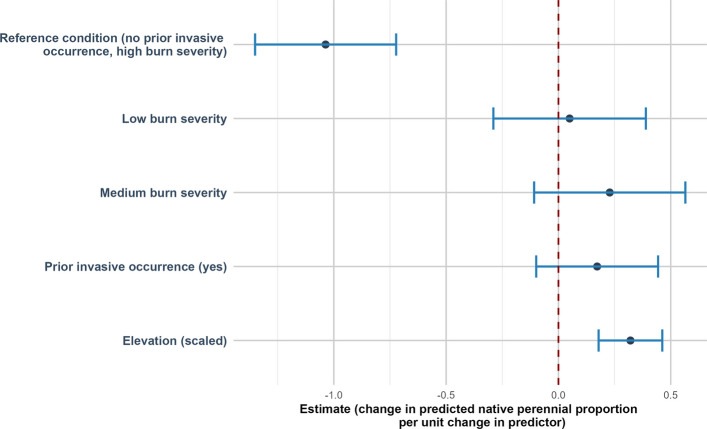
Direction and uncertainty of predictor effects on native perennial cover based on generalized linear mixed models. The reference condition represents predicted native perennial cover under high burn with no prior invasive occurrence, at mean-centered values of continuous predictors, and is shown for context only. Points indicate estimated model coefficients and horizontal bars represent 95% confidence intervals. Confidence intervals that do not overlap the vertical dashed zero line indicate statistically significant support for an effect, whereas intervals overlapping zero indicate weaker support

Native perennial (NP) cover was positively related to elevation (estimate of 0.32 (SE = 0.07, *p* < 0.001)) (Fig. 10).

## Discussion

Early post-wildfire vegetation patterns in the McKay Creek Wildfire area were not strongly associated with burn severity or documented pre-fire invasive plants occurrence when vegetation was considered at the broad status-group level (native or non-native). Predicted percent cover of native plants, non-native plants, and bare ground did not differ meaningfully across burn severity classes or between sites with and without documented pre-fire occurrence of invasive plants. Accordingly, our prediction that higher burn severity and documented pre-fire occurrence of invasive plants would correspond to higher post-fire non-native cover at the site was not supported 2 years after fire.

Although mean cover values for native plants, non-native plants, and bare ground were similar across fire severities and pre-fire occurrence of invasive plants and non-occurrence categories, mixed-effects models identified topography—especially elevation—as the key factor influencing early post-wildfire vegetation cover. Native cover increased with elevation, and non-native cover and bare ground both decreased. When plant groups were further examined by life cycle (annual, biennial, perennial), this pattern became more nuanced. Non-native annual cover was higher in high-severity burns than in low-severity burns and decreased with increasing elevation, and non-native perennials increased with elevation. These associations suggest that early post-fire vulnerability may be concentrated in low-elevation, high-severity sites, particularly for fast-establishing annuals, and non-native perennials may have an increased tolerance for harsher, high-elevation environments.

A central implication for early detection and rapid response (EDRR) of invasive plants in post-wildfire contexts is that early post-fire surveys are essential even when non-native plants are not yet abundant. Two features of this study—pre-fire invasive plant mapping and standardized early post-fire surveys—directly support EDRR by (1) identifying locations where invasion risk is highest immediately following disturbance and (2) establishing a baseline against which subsequent expansion, lagged establishment, and treatment outcomes can be evaluated. Importantly, the absence of a strong status-group signal at 2 years should not be interpreted as evidence of low invasion risk. Previous studies show that post-fire invasions can exhibit temporal lags with changes in species composition, dominance, and community structure emerging several years after initial post-fire recovery rather than immediately following disturbance (Abella et al. 2015, Weeks et al. 2023). For example, invasive annual grasses, such as cheatgrass, may remain sparse during the first few post-fire years but increase substantially with time since fire, suggesting that invasion pressure may increase as post-fire succession progresses (Shinneman and Baker 2009). In addition, small or sparse invasive plant infestations may remain undetected in coarse vegetation summaries while still acting as propagule sources for later spread, particularly across large or high-severity burn areas (Turner et al. 1997). From an EDRR perspective, early surveys may therefore represent the critical decision window in which prevention and rapid containment are most feasible and cost-effective, before invasive plant cover expands or reinforces fire-invasion feedbacks, particularly in sites where post-fire conditions favor rapid establishment by annual species (Simberloff et al. 2013; Brooks et al. 2004).

One explanation for why our prediction was not supported lies in the ecological mechanisms influencing plant recovery after wildfire. Fire influences post-disturbance recovery by altering both the physical environment, including soil properties, nutrient availability, and microclimate (Adkins et al. 2019; Lanta et al. 2025), and biological legacies such as surviving plants, seed banks, and soil microbial communities that constrain early vegetation responses (Pérez-Valera 2020, Pausas and Keeley 2014). For example, while it can eliminate seed banks in the upper soil layers, it may also stimulate the germination of deeply buried seeds (Santana et al. 2010; Roshan et al. 2022). These dynamics, combined with the survival and resprouting capabilities of perennial plants through rhizomes, may mask or override the effects of pre-fire occurrence of invasive plants or burn severity. Additionally, vegetation recovery is influenced by plant life-history strategies and regeneration traits that shape post-disturbance responses. Annual invasive plants often establish rapidly after disturbance due to fast germination, high growth rates, and prolific seed production under resource-rich conditions (Pyšek and Richardson 2007). Perennial species generally establish more slowly from seed but persist through longer-lived individuals and vegetative structures, resulting in slower population turnover and greater long-term persistence (Pyšek and Richardson 2007).

Given that our results contrast other findings, such as results from Yellowstone National Park that showed substantial effects of fire severity and existing invasive plant patch size on early post-fire plant cover and species (Turner et al. 2016), our study suggests that predictions regarding vegetation trajectories may not be widely applicable across differing landscapes. This difference may, in part, reflect the relatively early stage of recovery captured in our dataset. Several long-term studies have shown that vegetation responses to fire, particularly in relation to burn severity and invasive plants, often unfold over a longer time frame. For example, Shinneman and Baker (2009) documented that *B. tectorum* cover increased gradually over 7 to 8 years post-fire in semiarid ecosystems, peaking well after the initial 2-year period examined here. Similarly, Morgan et al. (2015) found that vegetation cover and species diversity remained suppressed on high-severity plots for at least 6 years following fire, highlighting the long-term influence of burn severity. Furthermore, Tepley et al. (2018) emphasize that high-severity burns can initiate fire–vegetation feedbacks that may not be detectable in early post-fire years but can eventually lead to landscape-scale shifts in vegetation composition. These studies collectively suggest that the muted effects of burn severity or previous occurrence of invasive plants observed in our study may reflect temporal lag rather than a lack of eventual impact, underscoring the need for long-term monitoring to fully understand post-fire vegetation trajectories. Another explanation for this discrepancy may lie in sampling design or the biophysical differences of the McKay Creek area. Our sampling plots were located away from roads, riparian corridors, and other disturbance vectors, potentially underrepresenting invasion hotspots and overrepresenting conditions less prone to non-native colonization. These contrasting findings highlight the importance of regionally specific, long-term monitoring of vegetation trajectories of post-wildfire landscapes to inform restoration.

Further limitations of our study must be acknowledged. While we benefited from strong pre-fire baseline data and high-resolution burn severity mapping, the scale and heterogeneity of the landscape limited sampling intensity. This low-intensity design, though spatially extensive, may have missed smaller-scale vegetation dynamics, rare species occurrences, or localized invasion hotspots. In addition, pre-fire invasive data were available primarily as mapped occurrence/extent records rather than plot-matched abundance by functional group, which limits our ability to test species or life-cycle-specific continuity (e.g., whether pre-fire non-native perennials predict post-fire perennial cover). Long-term monitoring with finer-resolution pre- and post-fire data would therefore be necessary to more fully evaluate continuity, lagged responses, and successional trajectories over time.

### Management and policy implications

Our results suggest that early post-fire monitoring and intervention should be prioritized by landscape position rather than burn severity alone. Elevation was the factor most strongly associated with early cover patterns on this fire at this stage of recovery, and life-cycle disaggregation indicated heightened vulnerability to non-native annual establishment in low-elevation, high-severity sites. From an EDRR perspective, these areas represent practical priorities for rapid post-fire surveys, because early infestations of non-native annual plants can be easier to contain before seed production and further spread.

More broadly, the lack of strong status-group contrasts across burn severity or pre-fire invasive plants occurrence reinforces the need for field-based early detection, not reliance on severity class or prior occurrence maps as sole screening tools. Pre-fire mapping remains valuable for targeting surveillance and evaluating change through time, but early post-fire decisions should be guided by on-the-ground confirmation and the recognition that invasion outcomes may emerge after lag periods. Given the increasing extent of land affected by wildfire, drone-based monitoring using high-resolution unmanned aerial vehicle (UAV) imagery and AI-assisted species identification may enable scalable detection across large, burned areas, particularly in high-severity sites where canopy loss increases ground visibility (Abeysinghe et al. 2019; Amarasingam et al. 2024; Bishop and Errigo 2023; Kattenborn et al. 2019).

Finally, because this study did not evaluate restoration treatments (e.g., seeding, planting, or invasive plant control), we avoid treatment-specific recommendations and instead situate management implications within established literature. Decisions regarding active intervention should be guided by site-specific objectives, propagule pressure, and local constraints. These decisions should be informed, where possible, by data-driven approaches grounded in early post-fire surveys, which can help identify areas of elevated risk and establish baselines for monitoring, prevention, and management interventions.

## Conclusion

This study offers new insights into the complex dynamics shaping post-wildfire vegetation recovery in British Columbia’s interior. Contrary to expectations, neither burn severity nor pre-fire occurrence of invasive plants emerged as strong predictors of post-fire vegetation outcomes. Instead, elevation played a dominant role in structuring native and non-native plant cover, underscoring the need to account for topographic variation in restoration planning. The relatively high proportion of native species observed suggests some resilience among fire-adapted communities, while the persistence of bare ground in burned areas identifies locations where vegetation cover may remain limited following fire and continued post-fire monitoring for native plant recovery and invasive plants occurrence could be prioritized.

As fire regimes intensify under climate change, region-specific studies focused on early post-fire conditions provide valuable baseline information for informing restoration efforts. Our results reinforce the importance of context-driven approaches and caution against assuming uniform responses to burn severity or prior invasion. Our continued commitment to long-term monitoring on the McKay Creek Wildfire and nearby fires on comparable landscapes will allow early patterns documented here to be evaluated in relation to longer-term vegetation trajectories in the future, with the hope of supporting evidence-based post-fire management in the region.

## Data Availability

Data sets generated during the current study are available from the corresponding author on reasonable request.

## References

[CR1] Abatzoglou, John T.., and A. Park. Williams. 2016. Impact of anthropogenic climate change on wildfire across Western US forests. *Proceedings of the National Academy of Sciences of the United States of America* 113 (42): 11770–11775. 27791053 10.1073/pnas.1607171113 PMC5081637

[CR2] Abella, Scott R.., and Paula J.. Fornwalt. 2015. Ten years of vegetation assembly after a North American mega fire. *Global Change Biology* 21 (2): 789–802. 10.1111/gcb.12722. 25200376 10.1111/gcb.12722

[CR3] Abeysinghe, Tharindu, Anita Simic Milas, Kristin Arend, et al. 2019. Mapping invasive *Phragmites australis* in the Old Woman Creek Estuary using UAV remote sensing and machine learning classifiers. *Remote Sensing *11 (11). 10.3390/rs11111380.

[CR4] Adkins, Jaron, Jonathan Sanderman, and Jessica Miesel. 2019. Soil carbon pools and fluxes vary across a burn severity gradient three years after wildfire in Sierra Nevada mixed-conifer forest. *Geoderma* 333 (January): 10–22. 10.1016/j.geoderma.2018.07.009.

[CR5] Alba, Christina, Hana Skálová, Kirsty F.. McGregor, Carla D’Antonio, and Petr Pyšek. 2015. Native and exotic plant species respond differently to wildfire and prescribed fire as revealed by meta-analysis. *Journal of Vegetation Science* 26 (1): 102–113. 10.1111/jvs.12212.

[CR6] Amarasingam, Narmilan, Fernando Vanegas, Melissa Hele, Angus Warfield, and Felipe Gonzalez. 2024. Integrating artificial intelligence and UAV-acquired multispectral imagery for the mapping of invasive plant species in complex natural environments. *Remote Sensing* 16 (9) : 1582. 10.3390/rs16091582.

[CR7] Applestein, Cara, Matthew J.. Germino, and Matthew R.. Fisk. 2018. Vegetative community response to landscape-scale post-fire herbicide (imazapic) application. *Invasive Plant Science and Management* 11 (3): 127–135. 10.1017/inp.2018.18.

[CR8] Archibald, S., C. E. R. Lehmann, C. M. Belcher, et al. 2018. Biological and geophysical feedbacks with fire in the Earth system. *Environmental Research Letters* 13 (3): 1–19. 10.1088/1748-9326/aa9ead.40201223

[CR9] Armstrong, Chelsey Geralda, Jennifer Grenz, Jennifer Zyp‐Loring, Jade LaFontaine, Leslie Main Johnson, and Nancy J.. Turner. 2025. Ethnoecological perspectives on environmental stewardship: tenets and basis of reciprocity in Gitxsan and Nłeʔkepmx (Nlaka’pamux) territories. *People and Nature* 7 (5): 934–946. 10.1002/pan3.10641.

[CR10] Balch, Jennifer K., Bethany A. Bradley, Carla M. D’Antonio, and José Gómez-Dans. 2013. Introduced annual grass increases regional fire activity across the arid Western USA (1980–2009). *Global Change Biology* 19 (1): 173–183. 10.1111/gcb.12046. 23504729 10.1111/gcb.12046

[CR11] Birthisel, Sonja Katherine, Ruth S. Clements, and Eric R. Gallandt. 2021. Review: how will climate change impact the ‘many little hammers’ of ecological weed management? *Weed Research* 61 (5): 327–341. 10.1111/wre.12497.

[CR12] Bishop, Tara B. B.., and Isabella M.. Errigo. 2023. Using sUAV imagery to map litter of invasive annual grass in dry environmental conditions. *Ecological Indicators* 146 (February) : 109755. 10.1016/j.ecolind.2022.109755.

[CR13] Bowman, David M. J. S.., Crystal A.. Kolden, John T.. Abatzoglou, Fay H.. Johnston, Guido R.. van der Werf, and Mike Flannigan. 2020. Vegetation fires in the Anthropocene. *Nature Reviews Earth & Environment* 1 (10): 500–515. 10.1038/s43017-020-0085-3.

[CR14] British Columbia Ministry of Forests. 2021. *2021 burn severity mapping (same year classification): methodology and data availability*. Victoria, BC: Forest Analysis and Inventory Branch.

[CR15] British Columbia Ministry of Forests. 2025. *Priority plants table – Province of British Columbia*. Province of British Columbia. Last modified December 16, 2025. https://www2.gov.bc.ca/gov/content/environment/plants-animals-ecosystems/invasive-species/priority-species/priority-plants/plants-table. Accessed March 2024.

[CR16] British Columbia Ministry of Forests and Range. 2010. *Invasive Alien Plant Program reference guide, part I*. Victoria, British Columbia, Canada: Range Branch.

[CR17] British Columbia Ministry of Forests. n.d. *Biogeoclimatic ecosystem classification: how BEC works*. https://www.for.gov.bc.ca/hre/becweb/system/how/index.html. Accessed March 2024.

[CR18] Brooks, Matthew L.., Carla M.. D’Antonio, David M.. Richardson, James B.. Grace, Jon E.. Keeley, Joseph M.. DiTomaso, Richard J.. Hobbs, Mike Pellant, and David Pyke. 2004. Effects of invasive alien plants on fire regimes. *BioScience* 54 (7): 677–688. 10.1641/0006-3568(2004)054[0677:EOIAPO]2.0.CO;2.

[CR19] Brooks, Mollie E.., Kasper Kristensen, Koen J.. van Benthem, Arni Magnusson, Casper W.. Berg, Anders Nielsen, Hans J.. Skaug, Martin Mächler, and Benjamin M.. Bolker. 2017. *Modeling zero-inflated count data with glmmTMB*. bioRxiv. 10.1101/132753.

[CR20] Canadian Interagency Forest Fire Centre (CIFFC). 2024. *Canada report: 2023 fire season*. Winnipeg, MB: Canadian Interagency Forest Fire Centre. https://www.ciffc.ca. Accessed 21 Dec 2025.

[CR21] Certini, Giacomo. 2005. Effects of fire on properties of forest soils: a review. *Oecologia* 143 (1): 1–10. 15688212 10.1007/s00442-004-1788-8

[CR22] Chambers, Jeanne C.., Richard F.. Miller, David I.. Board, David A.. Pyke, Bruce A.. Roundy, James B.. Grace, Eugene W.. Schupp, and Robin J.. Tausch. 2014. Resilience and resistance of sagebrush ecosystems: implications for state and transition models and management treatments. *Rangeland Ecology & Management* 67 (5): 440–454. 10.2111/REM-D-13-00074.1.

[CR23] Collins, L., K. Morrison, M. S. Buonanduci, et al. 2025. Extremely large fires shape fire severity patterns across the diverse forests of British Columbia, Canada. ARTICLE. *Ecosphere (Washington, United States)* 16 (8). 10.1002/ecs2.70364.

[CR24] Coop, Jonathan D.., Sean A.. Parks, Camille S.. Stevens-Rumann, Shelley D.. Crausbay, Philip E.. Higuera, Matthew D.. Hurteau, Alan Tepley, et al. 2020. Wildfire-driven forest conversion in western North American landscapes. *BioScience* 70 (8): 659–673. 32821066 10.1093/biosci/biaa061 PMC7429175

[CR25] D’Antonio, Carla M.., and Peter M.. Vitousek. 1992. Biological invasions by exotic grasses, the grass/fire cycle, and global change. *Annual Review of Ecology and Systematics* 23:63–87.

[CR26] Day, Nicola J.., Suzanne Carrière, and Jennifer L.. Baltzer. 2017. Annual dynamics and resilience in post-fire boreal understory vascular plant communities. *Forest Ecology and Management* 401 (October): 264–272. 10.1016/j.foreco.2017.06.062.

[CR27] Dickson-Hoyle, Sarah, Arial Eatherton, Jennifer N. Baron, Florencia Tiribelli, and Lori D. Daniels. 2024. Fire severity drives understory community dynamics and the recovery of culturally significant plants. ARTICLES. *Ecosphere (Washington, United States)* 15 (3). 10.1002/ecs2.4795.

[CR28] Dixon, Cinnamon M.., Kevin M.. Robertson, and Monica T.. Rother. 2024. Responses of plant species to mechanical soil disturbance in fire-dependent pine communities of the North American Coastal Plain - a synthesis. *Forest Ecology and Management* 565 (August) : 122018. 10.1016/j.foreco.2024.122018.

[CR29] Dukes, Jeffrey S.., Jennifer Pontius, David Orwig, Jeffrey R.. Garnas, Vikki L.. Rodgers, Nicholas Brazee, Barry Cooke, et al. 2009. Responses of insect pests, pathogens, and invasive plant species to climate change in the forests of northeastern North America: what can we predict? This article is one of a selection of papers from NE Forests 2100: a synthesis of climate change impacts on forests of the Northeastern US and Eastern Canada. *Canadian Journal of Forest Research* 39 (2): 231–248. 10.1139/X08-171.

[CR30] Environment and Climate Change Canada. 2024. *Canadian climate normals 1981–2010 station data—climate*. Retrieved September 15, 2024. https://climate.weather.gc.ca/climate_normals/results_1981_2010_e.html?stnID=960&autofwd=1.

[CR31] Flannigan, Mike, Alan S.. Cantin, William J.. de Groot, Mike Wotton, Alison Newbery, and Lynn M.. Gowman. 2013. Global wildland fire season severity in the 21st century. *Forest Ecology and Management, the Mega-Fire Reality* 294 (April): 54–61. 10.1016/j.foreco.2012.10.022.

[CR32] Fornwalt, Paula J.., and Merrill R.. Kaufmann. 2014. Understorey plant community dynamics following a large, mixed severity wildfire in a *Pinus ponderosa*–*Pseudotsuga menziesii* forest, Colorado, USA. *Journal of Vegetation Science* 25 (3): 805–818.

[CR33] Government of British Columbia. 2024. *Wildfire season summary*. https://www2.gov.bc.ca/gov/content/safety/wildfire-status/about-bcws/wildfire-history/wildfire-season-summary. Accessed March 2024.

[CR34] Grenz, Jennifer, and David R. Clements. 2023. Water, wind, and fire: extreme climate events enhance the spread of invasive plants in sensitive North American ecosystems. In *Plant invasions and global climate change*, edited by Sachchidanand Tripathi, Rahul Bhadouria, Priyanka Srivastava, Rishikesh Singh, and Daizy R. Batish, 113–37. Singapore: Springer Nature Singapore. 10.1007/978-981-99-5910-5_6.

[CR35] Hagmann, R. Keala., Jerry F.. Franklin, and K. Norman. Johnson. 2013. Historical structure and composition of ponderosa pine and mixed-conifer forests in south-central Oregon. *Forest Ecology and Management* 304 (September): 492–504. 10.1016/j.foreco.2013.04.005.

[CR36] Hagmann, R. K., P. F. Hessburg, S. J. Prichard, N. A. Povak, P. M. Brown, P. Z. Fulé, R. E. Keane, et al. 2021. Evidence for widespread changes in the structure, composition, and fire regimes of western North American forests. *Ecological Applications* 31 (8): 1–34. 10.1002/eap.2431 PMC9285092 34339067

[CR37] Harvey, Brian J., Daniel C. Donato, and Monica G. Turner. 2016. High and dry: post-fire tree seedling establishment in subalpine forests decreases with post-fire drought and large stand-replacing burn patches. *Global Ecology and Biogeography* 25 (5/6): 655–669.

[CR38] Hartig, Florian. 2022. *DHARMa: residual diagnostics for hierarchical (multi-level/mixed) regression models*. R package version 0.4.6.1. https://github.com/florianhartig/dharma.

[CR39] Harvey, Jill E.., Dan. J.. Smith, and Thomas T.. Veblen. 2017. Mixed-severity fire history at a forest? Grassland ecotone in west central British Columbia, Canada. *Ecological Applications* 27 (6): 1746–1760. 28434190 10.1002/eap.1563

[CR40] Heyerdahl, Emily K.., Ken Lertzman, and Carmen M.. Wong. 2012. Mixed-severity fire regimes in dry forests of southern interior British Columbia, Canada. *Canadian Journal of Forest Research* 42 (1): 88–98. 10.1139/x11-160.

[CR41] Integrated Taxonomic Information System. n.d. *Integrated Taxonomic Information System (ITIS) on-line database*. 10.5066/F7KH0KBK. Accessed March 2024.

[CR42] Inter-Ministry Invasive Species Working Group (IMISWG). 2014. *Invasive species early detection and rapid response plan for British Columbia*. Victoria, BC: Province of British Columbia.

[CR43] Invasive Species Council of BC. 2024a. *Spotted knapweed (Centaurea stoebe)*. https://bcinvasives.ca/invasives/spotted-knapweed/.

[CR44] Invasive Species Council of BC. 2024b. *Cheatgrass (Bromus tectorum)*. https://bcinvasives.ca/invasives/cheatgrass/.

[CR45] Johnstone, Jill F., Craig D. Allen, Jerry F. Franklin, Lee E. Frelich, Brian J. Harvey, Philip E. Higuera, Michelle C. Mack, et al. 2016. Changing disturbance regimes, ecological memory, and forest resilience. *Frontiers in Ecology and the Environment* 14 (7): 369–378.

[CR46] Jones, Vanessa L., and Jennifer Grenz. 2023. “A review of the impacts and management of invasive plants in forestry.” *CABI Reviews* 2023. 10.1079/cabireviews.2023.0034.

[CR47] Kattenborn, Teja, Javier Lopatin, Michael Förster, Andreas Christian Braun, and Fabian Ewald Fassnacht. 2019. UAV data as alternative to field sampling to map woody invasive species based on combined Sentinel-1 and Sentinel-2 data. *Remote Sensing of Environment* 227 (June): 61–73. 10.1016/j.rse.2019.03.025.

[CR48] Keeley, Jon E. 2006. Fire management impacts on invasive plants in the Western United States. *Conservation Biology* 20 (2): 375–384. 16903098 10.1111/j.1523-1739.2006.00339.x

[CR49] Klinkenberg, Brian, ed. n.d. *E-Flora BC: electronic atlas of the flora of British Columbia*. Lab for Advanced Spatial Analysis, Department of Geography, University of British Columbia. https://linnet.geog.ubc.ca/biodiversity/eflora/index.html. Accessed March 2024.

[CR50] Lanta, Vojtěch, Martin Adámek, Zuzana Chlumská, et al. 2025. Plant colonisation, soil nutrient patterns and microclimate after a large forest fire in temperate Central Europe. *Forest Ecology and Management* 585 (June) : 122643. 10.1016/j.foreco.2025.122643.

[CR51] Leung, Corrie. 2002. *Invasion of British Columbia’s grasslands*. Vancouver, Canada: Canadian Parks and Wilderness Society BC Chapter.

[CR52] Lewis, Michael, Amy Christianson, and Marsha Spinks. 2018. Return to flame: reasons for burning in Lytton First Nation, British Columbia. *Journal of Forestry* 116 (2): 143–150. 10.1093/jofore/fvx007.

[CR53] MacKenzie, William H.., and Del V.. Meidinger. 2018. The biogeoclimatic ecosystem classification approach: an ecological framework for vegetation classification. *Phytocoenologia* 48 (2): 203–213. 10.1127/phyto/2017/0160.

[CR54] Magnusson, Arni, Hans J. Skaug, Anders Nielsen, Casper W. Berg, Kristensen Kasper, Martin Maechler, Koen J. van Bentham, Benjamin M. Bolker, and Mollie E. Brooks. 2017. *glmmTMB: generalized linear mixed models using template model builder*. R package version 0.1.3. https://github.com/glmmTMB.

[CR55] Meidinger, D., and J. Pojar. 1991. *Ecosystems of British Columbia*. Special report series/British Columbia. Ministry of Forests; No. 6 ISSN 0843–6452. Victoria: Ministry of Forests.

[CR56] Merriam, Kyle E.., Jon E.. Keeley, and Jan L.. Beyers. 2006. Fuel breaks affect nonnative species abundance in Californian plant communities. *Ecological Applications* 16 (2): 515–527. 16711041 10.1890/1051-0761(2006)016[0515:fbansa]2.0.co;2

[CR57] Michalcová, Dana, Samuel Lvončík, Milan Chytrý, and Ondřej Hájek. 2011. Bias in vegetation databases? A comparison of stratified-random and preferential sampling. *Journal of Vegetation Science* 22 (2): 281–291.

[CR58] Morgan, Penelope, Marshell Moy, Christine A.. Droske, Sarah A.. Lewis, Leigh B.. Lentile, Peter R.. Robichaud, Andrew T.. Hudak, and Christopher J.. Williams. 2015. Vegetation response to burn severity, native grass seeding, and salvage logging. *Fire Ecology* 11 (2): 31–58. 10.4996/fireecology.1102031.

[CR59] National Invasive Species Council (NISC). 2005. *Five-year review of Executive Order 13112 on invasive species*. https://digitalcommons.unl.edu/natlinvasive/17.

[CR60] Neary, Daniel G., Carole C. Klopatek, Leonard F. DeBano, and Peter F. Ffolliott. 1999. Fire effects on belowground sustainability: a review and synthesis. *Forest Ecology and Management* 122 (1): 51–71. 10.1016/S0378-1127(99)00032-8.

[CR61] Oeggerli, Virginia, and Jennifer Grenz. 2025. From science to sovereignty: indigenizing western scientific approaches for culturally appropriate wildfire recovery. RESEARCH ARTICLE. *Ecology and Evolution (Bognor Regis, United States)* 15 (8). 10.1002/ece3.71939.

[CR62] Parks, Sean A.., Carol Miller, Marc-André. Parisien, Lisa M.. Holsinger, Solomon Z.. Dobrowski, and John Abatzoglou. 2015. Wildland fire deficit and surplus in the Western United States, 1984–2012. *Ecosphere* 6 (12): 1–13. 10.1890/ES15-00294.1.

[CR63] Pausas, Juli G., and Jon E. Keeley. 2014. Evolutionary ecology of resprouting and seeding in fire-prone ecosystems. *The New Phytologist* 204 (1): 55–65. 25298997 10.1111/nph.12921

[CR64] Pérez-Valera, E., M. Verdú, J. A. Navarro-Cano, and M. Goberna. 2020. Soil microbiome drives the recovery of ecosystem functions after fire. *Soil Biology & Biochemistry* 149 (October) : 107948. 10.1016/j.soilbio.2020.107948.

[CR65] Pyšek, Petr, and David M. Richardson. 2007. *Traits associated with invasiveness in alien plants: where do we stand?* In Biological invasions, edited by Wolfgang Nentwig. Springer. 10.1007/978-3-540-36920-2_7.

[CR66] Ricci, Lorenzo, Beatrice Farda, Arianna Ferrara, et al. 2024. Short-term functional response to post-fire vegetation dynamic: a case study in a Mediterranean Pinus halepensis forest. *Flora* 310 (January) : 152415. 10.1016/j.flora.2023.152415.

[CR67] Richardson, David M., and Petr Pyšek. 2004. *What is an invasive species?* Available online at http://www.cabicompendium.org/cpc/aspects.asp?.

[CR68] Roshan, Sina Attar, Mehdi Heydari, Alexander Wait, S. M. Mijan. Uddin, Manuel Esteban Lucas-Borja, and Jon E.. Keeley. 2022. Divergent successional trajectories of soil seed bank and post-fire vegetation in a semiarid oak forest: implications for post-fire ecological restoration. *Ecological Engineering* 182 (September) : 106736. 10.1016/j.ecoleng.2022.106736.

[CR69] Santana, Victor M.., Ross A.. Bradstock, Mark K. J.. Ooi, Andrew J.. Denham, Tony D.. Auld, and M. Jaime. Baeza. 2010. Effects of soil temperature regimes after fire on seed dormancy and germination in six Australian Fabaceae species. *Australian Journal of Botany* 58 (7): 539–545. 10.1071/BT10144.

[CR70] Sayedi, Sayedeh Sara, Benjamin W.. Abbott, Boris Vannière, Bérangère. Leys, Daniele Colombaroli, Graciela Gil Romera, Michał Słowiński, et al. 2024. Assessing changes in global fire regimes. *Fire Ecology* 20 (1) : 18. 10.1186/s42408-023-00237-9.

[CR71] Shephard, Noah T.., Lana Narine, Yucheng Peng, and Adam Maggard. 2022. Climate smart forestry in the Southern United States. *Forests* 13 (9) : 1460. 10.3390/f13091460.

[CR72] Shinneman, Douglas J.., and William L.. Baker. 2009. Environmental and climatic variables as potential drivers of post-fire cover of cheatgrass (*Bromus tectorum*) in seeded and unseeded semiarid ecosystems. *International Journal of Wildland Fire* 18 (2): 191–202. 10.1071/WF07043.

[CR73] Shryock, Daniel F.., Lesley A.. DeFalco, and Todd C.. Esque. 2014. Life-history traits predict perennial species response to fire in a desert ecosystem. *Ecology and Evolution* 4 (15): 3046–3059. 10.1002/ece3.1159. 25247062 10.1002/ece3.1159 PMC4161178

[CR74] Simberloff, Daniel, Jean-Louis. Martin, Piero Genovesi, et al. 2013. Impacts of biological invasions: what’s what and the way forward. *Trends in Ecology & Evolution* 28 (1): 58–66. 10.1016/j.tree.2012.07.013. 22889499 10.1016/j.tree.2012.07.013

[CR75] Still, C. J., A. Sibley, D. DePinte, P. E. Busby, C. A. Harrington, M. Schulze, D. R. Shaw, et al. 2023. Causes of widespread foliar damage from the June 2021 Pacific Northwest heat dome: more heat than drought. *Tree Physiology* 43 (2): 203–209. 10.1093/treephys/tpac143. 36611006 10.1093/treephys/tpac143

[CR76] Tepley, Alan J.., Enrique Thomann, Thomas T.. Veblen, George L. W.. Perry, Kristina J.. Anderson-Teixeira, Andrés Holz, Juan Paritsis, and Thomas Kitzberger. 2018. Influences of fire-vegetation feedbacks and post-fire recovery rates on forest landscape vulnerability to altered fire regimes. *Journal of Ecology* 106 (5): 1925–1940.

[CR77] Turner, Monica G.., William H.. Romme, Robert H.. Gardner, and William W.. Hargrove. 1997. Effects of fire size and pattern on early succession in Yellowstone National Park. *Ecological Monographs* 67 (4): 411–433. 10.1890/0012-9615(1997)067[0411:EOFSAP]2.0.CO;2.

[CR78] Turner, Monica G.., Timothy G.. Whitby, Daniel B.. Tinker, and William H.. Romme. 2016. Twenty-four years after the Yellowstone fires: are postfire lodgepole pine stands converging in structure and function? *Ecology* 97 (5): 1260–1273. 27349102 10.1890/15-1585.1

[CR79] U.S. Department of Agriculture, Forest Service. n.d. *Fire Effects Information System (FEIS)*. Rocky Mountain Research Station, Missoula Fire Sciences Laboratory. https://www.feis-crs.org/feis/. Accessed March 2024.

[CR80] Weeks, Jonah Maria, Jesse E. D.. Miller, Zachary L.. Steel, Evan E.. Batzer, and Hugh D.. Safford. 2023. High-severity fire drives persistent floristic homogenization in human-altered forests. *Ecosphere* 14 (2) : e4409. 10.1002/ecs2.4409.

[CR81] Zouhar, Kristin, Jane Kapler Smith, and Steve Sutherland. 2008. *Effects of fire on nonnative invasive plants and invasibility of wildland ecosystems*. In Wildland fire in ecosystems: fire and nonnative invasive plants, edited by Kristin Zouhar, Jane Kapler Smith, Steve Sutherland, and Matthew L. Brooks, 7–32, 42. Gen. Tech. Rep. RMRS-GTR-42, vol. 6. Ogden, UT: U.S. Department of Agriculture, Forest Service, Rocky Mountain Research Station.

